# Effects of whole-body electromyostimulation with different impulse intensity on blood pressure changes in hyper- and normotensive overweight people. A pilot study

**DOI:** 10.3389/fphys.2024.1349750

**Published:** 2024-02-22

**Authors:** Wolfgang Kemmler, Matthias Kohl, Simon von Stengel, Sebastian Willert, Stephanie Kast, Michael Uder

**Affiliations:** ^1^ Institute of Radiology, University Hospital Nürnberg, Erlangen, Germany; ^2^ Institute of Medical Physics, Friedrich-Alexander-University of Erlangen-Nürnberg, Erlangen, Germany; ^3^ Faculty Medical and Life Sciences, University of Furtwangen, Villingen-Schwenningen, Germany

**Keywords:** whole-body electromyostimulation, neuromuscular electrical stimulation, blood pressure, stimulus intensity, impulse intensity, hypertension, cardiovascular

## Abstract

Hypertension is a frequent condition in untrained middle-aged to older adults, who form the core group of whole-body electromyostimulation (WB-EMS) applicants. So far, the acute effects of varying impulse intensities on blood pressure responses have not been evaluated in normo- and hypertensive people. Thirteen hypertensive and twelve normotensive overweight WB-EMS novices, 40–70 years old, conducted the same WB-EMS protocol (20 min, bipolar, 85 Hz, 350 µs, 4 s impulse-4 s rest; combined with easy movements) with increasing impulse intensity (low, moderate, advanced) per session. Mean arterial blood pressure (MAP) as determined by automatic sphygmomanometry rose significantly (*p* < .001) from rest, 5 min pre-WB-EMS to immediately pre-WB-EMS assessment. Of importance, a 20-min WB-EMS application does not increase MAP further. In detail, maximum individual MAP does not exceed 128 mmHg (177 mmHg systolic or 110 mmHg diastolic) in any case. Two-min post-WB-EMS, MAP was significantly lower (*p* = .016) compared to immediately pre-WB-EMS. In contrast, heart rate increased significantly from immediately pre to immediately post-exercise (*p* < .001), though individual peak values did not exceed 140 beats/min^−1^ and heart rate decreased rapidly (*p* < .001) post-exercise. No significant differences in MAP and HR kinetics were observed for impulse intensity categories or hypertensive status. In summary, largely independently of impulse intensity and status, the acute effect of WB-EMS on MAP in novice applicants seem to be largely negligible. Although definite evidence might not have been provided by the present study, we conclude that hypertension, at least under treatment, should not be considered as a barrier for WB-EMS application in moderately old or older cohorts.

## 1 Introduction

WB-EMS is an increasingly popular exercise technology predominantly applied to address physical function, body composition and health-related outcomes. Due to its joint friendliness, time-efficiency and close supervision, WB-EMS might be a particular attractive training method for people unable or unmotivated to conduct conventional exercise programs. Although the safe application of WB-EMS is specified by (albeit non-mandatory) guidelines (Kemmler et al., 2023) and contraindications (Kemmler et al., 2019), several issues on safe WB-EMS application remain. In its predominately applied specification, WB-EMS is often considered as more of a resistance type exercise (Kemmler et al., 2022). In this context, studies on acute effects of intense resistance exercise/weightlifting on arterial blood pressure [e.g., (MacDougall et al., 1985)] revealed very pronounced peak pressures, incompatible with health-related outcomes in non-athletic cohorts. This aspect might have contributed to the decision to deem at least untreated hypertension (Kemmler et al., 2019) an absolute contraindication for commercial, non-medical WB-EMS. To our best knowledge, apart from a few studies that reported results of acute effects of WB-EMS on blood pressure (BP) as a secondary study outcome (Hoshiai et al., 2021; Cassemiliano et al., 2022; Alvarez-Barrio et al., 2023), no further study has focused on blood pressure changes related to WB-EMS. The fact that the majority of clients of the large commercial German WB-EMS facilities are middle aged to older people (EMS-Training.de, 2017), i.e., a cohort with a high prevalence of hypertension (Neuhauser et al., 2016; Neuhauser et al., 2017), illustrates the relevance of providing evidence on acute WB-EMS induced effects on BP in middle aged-older people. Of further importance, due to the limited sensitivity to endogenous electrical stimuli, the first few weeks of WB-EMS application might be particularly critical for adverse effects due to errors in intensity specification (Teschler et al., 2016). The aim of the present pilot study was thus to determine the immediate effect of WB-EMS on mean arterial pressure (MAP) by applying different stimulus intensities in hypertensive and normotensive middle aged to older novice applicants during the first 4 weeks of WB-EMS. Based on occasional assessments of acute BP in other WB-EMS studies (Kemmler et al., 2010; Kemmler et al., 2018; Kemmler and von Stengel, 2020) our primary hypothesis was that 20 min of conventional WB-EMS application would not significantly increase MAP largely independently of the stimulus intensity. Our secondary hypothesis was that both cohorts, people with and without hypertension, show comparable WB-EMS^1^-induced MAP changes. Finally, an experimental hypothesis was that heart rate increases significantly due to standard WB-EMS combined with easy movements but quickly returns to baseline values post exercise.

## 2 Methods

The Franconian EMS and arterial blood pressure study (FrEMAP) study is a cross-sectional WB-EMS study that aimed to determine the effects of WB-EMS combined with easy movements with different stimulus intensities on MAP in novice applicants with and without hypertension. The study included men and women 40–70 years old, with overweight and osteoarthritis of the knee. The project was designed and initiated by the Institute of Radiology, University Hospital Erlangen (UKER) Germany. The University Ethics Committee approved the trial (number 359_19b). The study fully complies with the Helsinki Declaration (World Medical, 2013). After receiving detailed information, all study participants gave their written informed consent.

### 2.1 Study design

**FIGURE 1:**
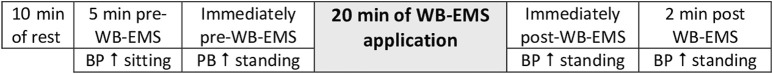
Schedule of blood pressure assessments during the WB-EMS session.

The present study compared the effects of three standardized WB-EMS sessions with different stimulus intensities (low vs. moderate vs. advanced) on blood pressure changes in novice WB-EMS applicants. Comparable to the conventional, commercial approach, after a familiarization session, impulse intensity of the WB-EMS application was linearly increased during the subsequent sessions in order to reach an adequate stimulus intensity after 4–6 weeks. In detail, we specified a low-moderate stimulus intensity (i.e., 4 on Borg CR-10 scale) for the second session, a moderate stimulus intensity (i.e., 5 on Borg CR-10 scale) for the third session and an advanced stimulus intensity (i.e., 6-<7 on Borg CR-10 scale) for the fourth session. Resting blood pressure and HR was determined in a sitting position after 10 min of rest and 5 min pre-WB-EMS (5′pre-WB-EMS) and immediately (<5 s) before (pre-WB-EMS), immediately (<5 s) after 20 min (post-WB-EMS) and 2 min post-WB-EMS application (2′post-WB-EMS) each in a standing position (Figure 1).

### 2.2 Participants

Participants were former members of the control group of the “Whole-body Electromyostimulation for the Treatment of Knee Osteoarthritis” (EMSOAT) trial, a randomized controlled trial that focused on the effects of WB-EMS on knee arthrosis (clinical trials.gov: NCT05672264). Briefly, eligibility criteria of EMSOAT with relevance for the present topic were: (a) men or women 40–70 years old, with (b) overweight (BMI>25 kg/m^2^). (c) Femurotibial osteoarthritis with (d) osteoarthritic knee pain for at least 3 months. The EMSOAT protocol excluded people with (a) strength training for more than 60 min per week in the last year. (b) Glucocorticoid or opioid medication (c) Conditions and diseases (e.g., rheumatoid arthritis, fibromyalgia). with relevant impact on outcomes related to osteoarthritis of the knee, inflammation or contraindications for WB-EMS application [e.g., electric implants, epilepsy, cardiac pacemakers (Kemmler et al., 2019)]. Thirty-six participants randomly assigned to the non-exercising control group of the EMSOAT study (CG) were invited to participate in a 4-week WB-EMS program after the EMSOAT study period^2^. Twenty-eight participants took up our offer; finally thirteen hypertensive^3^ and twelve normotensive participants agreed to participate and were included in the present study.

### 2.3 Study intervention

We applied the conventional WB-EMS (combined with easy movements)^4^ protocol predominantly used in scientific and commercial settings (Beier et al., 2024). This approach focuses on a careful increase of stimulation intensity during the subsequent sessions after a familiarization session. Following the “updated international guideline for safe and effective whole-body” (Kemmler et al., 2023), we applied a once weekly 20 min session of WB-EMS in this cohort of WB-EMS novices. Briefly, we used the recognized (Beier et al., 2024) miha bodytec^®^ Type II (Gersthofen, Germany) equipment. In detail, ten main muscle groups (both thighs, gluteal muscles, abdomen, chest, lower back, latissimus area, upper back, both upper arms) were simultaneously stimulated with dedicated intensity for each region. Bipolar electric current with a frequency of 85 Hz, an impulse-width of 350 µs, and a rectangular pulse wave was applied during the 6 s impulse phase intermitted by 4 s of impulse break. During the impulse phase, low-intensity movements without any additional weight were performed in a standing position. Each session contains 6 trunk-specific movements [low-amplitude squat with latissimus pulleys, butterfly reverse (with angled arms), straight pullovers with trunk flexion, standing trunk flexion (crunch), one-legged stand (extended knee) with biceps curl, side step with weight shift and biceps curl (Weissenfels et al., 2019)] structured in 3 sets of 6 repetitions. Participants were asked to perform the movements/exercises without relevant effort. In order to strictly standardize the WB-EMS procedure, the sessions were video-guided. Exercise intensity of the combined WB-EMS/low intensity movement approach was specified based on the rate of perceived exertion (RPE) of the participant. The specification was roughly based on the Borg CR-10 scale (Borg and Borg, 2010) to schedule the stimulus intensity. Briefly (impulse), intensity was individually adapted for each body region in close interaction (1 instructor: 2 participants) (Kemmler et al., 2023) between the instructor and the participant during the first session. The settings were saved on a chip card to enable a quick start at the beginning of the next session. During the session, instructors adapted (impulse) intensity every 3 min in close cooperation with the participants to achieve and maintain the prescribed RPE during the session.

### 2.4 Study outcomes

#### 2.4.1 Primary study outcome

• Changes of acute mean arterial blood pressure (MAP).

#### 2.4.2 Secondary outcomes

• Changes of acute heart rate (HR).

### 2.5 Assessments

Great emphasis was placed on the standardization of the tests. All participants were requested to refrain from intense physical activity and exercise 48 h and to refrain from coffee or tea for at least 2 h prior to testing. The same experienced researcher used the same identically calibrated devices in exactly the same setting and at the same time of day (10:00 a.m. ± 30 min). In parallel, the same instructor consistently performed the WB-EMS application.

#### 2.5.1 Baseline characteristics

Height was determined with a stadiometer (Holtain Ltd. Crymmych, Wales), weight, and body composition were assessed via multi-frequent bioelectrical impedance analysis (DSM-BIA, InBody770, BioSpace, Seoul, Korea).

#### 2.5.2 Blood pressure and heart rate

After 10 min of relaxation, blood pressure (BP) and heart rate (HR) was determined with an automatic sphygmomanometer (Bosco, Bosch, Jungingen, Germany) twice on the right arm with a rest of 20 s between the samples (a) in a sitting position 5 min before exercise (5′pre-WB-EMS). (b) The second MAP assessment was conducted after 5 min in a standing position, immediately pre-WB-EMS-application (pre-WB-EMS). The third MAP assessment was conducted immediately post-20 min WB-EMS (post-WB-EMS), and finally (d) 2 min post-exercise (2′post-WB-EMS) in a standing position (Figure 1). MAP was calculated using the formula (diastolic BP + diastolic BP + systolic BP)/3.

### 2.6 Validation of impulse intensity

During the first session, participants were carefully briefed how to apply the RPE 10 scale. Participants were asked to exercise with low to moderate RPE during the second session, moderate RPE during the third session and advanced (“higher”) intensity during the third session. In close interaction between participants and instructor, the intended stimulus intensity/RPE specification was generated and then recorded at the end of the session. In order to validate RPE-based stimulus intensity we also recorded the stimulus intensity provided by the device for each of the three sessions. We compared RPE reported by the participants and impulse intensity data recorded by the device in order to ensure the increasing intensity during the three sessions.

### 2.7 Statistical analysis

We applied an Intention to treat (ITT) approach that included all participants who started WB-EMS application regardless of their compliance or confounding aspects. We applied multiple imputation of missing values (see below) using R statistics software (4.3.2 Patched; R Development Core Team Vienna, Austria) in combination with Amelia II (Honaker et al., 2011). We used the full data set for multiple imputations, and repeated imputation 100 times. Differences between stimulus intensity, hypertension categories and MAP changes were analyzed with linear mixed-effects models. Briefly, we used a linear mixed-effects model with random intercept. Differences between stimulus intensity, hypertension categories and MAP changes were analyzed with linear mixed-effects models where these variables were included as fixed-effects in addition to random intercepts for subjects. Post hoc tests with *p*-values adjusted according to the method of Holm (Holm, 1979) were applied to check differences between the MAP assessments (i.e., 5′pre-WB-EMS, pre-WB-EMS, post-WB-EMS, 2′post-WB-EMS). All tests were 2-tailed, significance was accepted at (adjusted) *p* < 0.05.

## 3 Results

### 3.1 Baseline characteristics

Table 1 displays baseline characteristics of the study participants. Briefly, 15 women and 10 men were included in the study. As per the eligibility criteria, participants were overweight to obese. Thirteen participants suffered from hypertension, 12 were normotensive. Of importance, two of the 13 hypertensive participants were not under pharmacologic therapy.

**TABLE 1:** Baseline characteristic of the cohort comprised of 15 women and 10 men with (*n* = 13) and without (*n* = 12) hypertension. MV±SD: means values ± standard deviations.

	Normotension MV±SD	Hypertension MV±SD
Gender (women/men) [*n*]	7/5	8/5
Age [years]	55.2 ± 6.7	59.3 ± 7.4
Body height [cm]	171.3 ± 7.3	178.4 ± 10.3
Body mass [kg] ^a^	85.8 ± 12.9	93.1 ± 13.6
Waist circumference [cm]	96.0 ± 10.3	102.3 ± 10.6
Body fat rate [%]^a^	35.4 ± 8.7	32.2 ± 7.6
Physical activity [Index] ^b^ ^,^ ^c^	4.1 ± 1.2	3.7 ± 0.8
Exercise (≥1 session per week) [*n*] ^c^	7 (58%)	8 (62%)
Three and more diseases [*n*] ^d^	2 (17%)	3 (23%)
Active Smokers [*n*] ^b^	3 (25%)	3 (23%)

^a^ As determined by BIA.

^b^ Scale: 1 = very low to 7 = very high.

^c^ Participant reported.

^d^ Confirmed by medical records.

### 3.2 Adherence to the protocol, unintended side effects

Twenty participants conducted all WB-EMS sessions while three participants missed the low-intensity session and one participant each was unable to visit the moderate or advanced intensity WB-EMS session. Reviewing the participants’ self-reported exercise intensity, compliance with the protocol was satisfactory. In detail, average RPE on the Borg CR10 was rated 4.8 ± 0.4 RPE for the low-moderate intensity session (specification “4”), 5.1 ± 0.6 for the moderate stimulus intensity session (specification “5”) and RPE 6.0 ± 0.3 (specification “6-<7”) for the fourth, i.e., advanced intensity session. Records provided by the device revealed that all participants increased absolute stimulus intensity from session one to three^5^. In detail, average impulse intensity was 62% ± 9% (first session), 69% ± 7% (second session) and 74% ± 7%^6^ (third session). No adverse effect was observed during the WB-EMS/low-intensity movement application; moreover, apart from muscular soreness no negative effects were reported after the WB-EMS session.

### 3.3 Study outcomes

**FIGURE 2:**
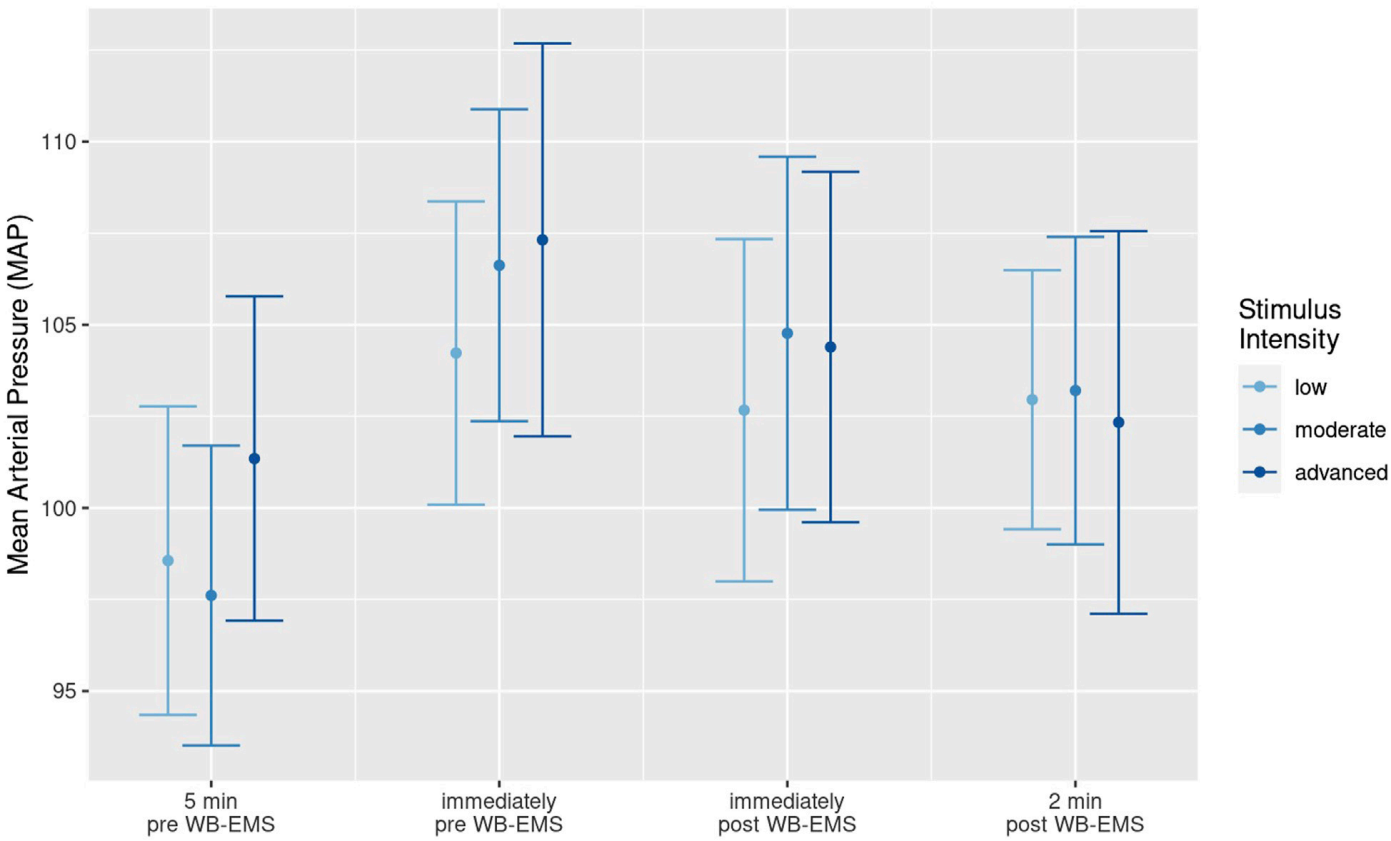
Mean value and 95%-CI for Mean Arterial Pressure (MAP) before (5′pre-, immediately pre-WB-EMS) and after (immediately post and 2′post-WB-EMS) WB-EMS application with low, moderate and advanced stimulus intensity.

We observed no (significant) differences in MAP and heart rate between genders or between participants ≤55 years vs. >55 years, so we conducted a joint analysis. Figure 2 displays WB-EMS induced changes of MAP during low, moderate and advanced impulse intensity for the total group (n = 25). Applying the linear-mixed effects model, we do not observe significant differences of MAP changes between the low vs. moderate vs. advanced impulse intensity approach (*p* = .390). In parallel, no significant differences were observed between normotensive versus hypertensive (*p* = .567) participants. On the other hand, average MAP changed significantly over the course of time (*p* < .001). Following a significant average rise in MAP from the resting sitting position 5′pre-WB-EMS to immediate pre-WB-EMS (6.9 mmHg; adj. *p* < .001), we observed a non-significant MAP reduction from pre-to immediately post- (−1.9 mmHg, adj *p* = .20) and a significant decrease pre to 2′post-WB-EMS (−3.3 mmHg, adj. *p* = .016). Individual MAP was maintained or decreased “during WB-EMS” (i.e., immediately pre-vs. post-WB-EMS) in 17 (advanced intensity) to 19 (moderate intensity) participants respectively. Relevant WB-EMS (≥10 mmHg) increases in MAP were observed in two normotensive participants, with a maximum rise of 16 mmHg during the advanced impulse intensity condition. In parallel, absolute MAP after WB-EMS did not exceed 128 mmHg (or 177 mmHg systolic BP and 110 mmHg diastolic BP) in any case^7^. Thus, in summary, 20 min of conventional WB-EMS application did not significantly increase MAP in normo- and hypertensive applicants largely independently of stimulus intensity and BP status.

Table 2 presents the raw values of HR. After a rise of HR from 5′pre to pre-WB-EMS (5.3 bpm, adj. *p* < .001), HR significantly increased (16.6 bpm, adj. *p* < .001) between immediately pre- and post-exercise and rapidly decreased (9.7 bpm, adj. *p* < .001) after WB-EMS (2′post). In parallel to MAP, we did not observe significant differences for HR changes between impulse intensity (*p* = .588) and hypertension categories (*p* = .578) (Table 2). Reviewing individual HR peaks, the highest value recorded after WB-EMS (140 bpm) was for a hypertensive participant after advanced intensity stimulation. Thus, we also confirmed our experimental hypothesis that heart rate increases significantly between immediately pre and post-exercise but quickly returns to baseline values post exercise.^8^

**TABLE 2:** Mean value and standard deviation for Mean Arterial Pressure (MAP) before (5′pre-, immediately pre-WB-EMS) and after (immediately post and 2′post) WB-EMS application with low, moderate and advanced impulse intensity. Total group (*n* = 25)^8^, normotensive (*n* = 12) and hypertensive subgroup (*n* = 13). Shaded areas: WB-EMS application.

Group	Low impulse intensity				Moderate impulse intensity				Advanced impulse intensity			
Mean arterial blood pressure (MAP) [mmHg]												
Time	5′pre-WB-EMS Rest, sitting	Immediately pre-WB-EMS standing	Immediately post-WB-EMS standing	2′post-WB-EMS, standing	5′pre-WB-EMS Rest, sitting	Immediately pre-WB-EMS standing	Immediately post-WB-EMS standing	2′post-WB-EMS, standing	5′pre-WB-EMS Rest, sitting	Immediately pre-WB-EMS standing	Immediately post-WB-EMS standing	2′post-WB-EMS, standing
Normotensive	95.6 ± 9.9	100.0 ± 11.0	101.7 ± 13.6	101.4 ± 11.3	95.0 ± 8.6	103.3 ± 10.5	103.8 ± 12.5	103.1 ± 12.6	102.0 ± 13.1	108.0 ± 14.8	107.0 ± 13.1	105.9 ± 12.1
Hypertensive	101.0 ± 8.3	107.8 ± 6.1	103.4 ± 7.7	104.3 ± 3.7	100 ± 9.9	109.7 ± 8.6	105.7 ± 10.2	103.3 ± 6.7	100.8 ± 7.2	106.1 ± 11.6	102.0 ± 8.7	99.1 ± 11.6

Heart rate (beats min-1; bpm)												
Total group	77.0 ± 10.2	82.8 ± 13.0	97.5 ± 20.1	90.1 ± 14.3	78.0 ± 10.6	85.4 ± 11.5	99.2 ± 18.4	89.7 ± 15.7	79.1 ± 11.5	82.9 ± 13.3	102.3 ± 16.7	89.6 ± 18.5
Normotensive	77.2 ± 8.9	85.4 ± 11.5	98.0 ± 11.8	89.3 ± 12.8	80.6 ± 10.7	86.6 ± 12.4	104.5 ± 14.9	92.4 ± 10.3	78.0 ± 11.7	82.6 ± 14.1	102.6 ± 14.2	90.9 ± 16.6
Hypertensive	76.9 ± 11.6	80.7 ± 11.6	97.1 ± 23.2	92.3 ± 15.8	75.6 ± 10.3	84.3 ± 10.9	94.3 ± 20.5	87.3 ± 19.6	80.1 ± 13.3	83.2 ± 13.3	102.1 ± 19.5	88.5 ± 20.8

## 4 Discussion

In summary, from our preliminary results, the acute effect of WB-EMS combined with easy movements on mean arterial blood pressure can be rated negligible to low. Indeed our finding of slight MAP increases with discreet average and individual peaks of systolic and diastolic BP values (that did not exceed 128 mmHg (MAP) or 177/110 mmHg in any case), indicates the low risk of WB-EMS triggering hypertensive effects. As hypothesized, this result is widely independent of stimulus intensity and BP status (i.e., hyper-vs. normotensive). In parallel, the physiologically less pronounced average HR increase of about 20 bpm with an individual maximum of 140 bpm and a rapid decrease towards initial HR values 2 min post-WB-EMS confirmed the low cardiovascular stress of WB-EMS application. The finding that changes in MAP were even more affected by the shift from the resting (5′pre) to the standing position compared with the change from immediately pre-to immediately post exercise, quite independently of stimulus intensity and BP status, might be the most striking evidence for applicability in cohorts with (treated) hypertension. The latter estimation was further underpinned by the finding that the discreet MAP-lowering effect of WB-EMS was more evident in participants with hypertension.

While studies that applied local NMES application were not applicable, only a few other studies addressed acute WB-EMS-induced changes of hemodynamic parameters as a study outcome [e.g., (Hoshiai et al., 2021; Cassemiliano et al., 2022)]. In summary however, no WB-EMS study reported significant changes of BP or heart rate. Closest to our WB-EMS approach, Hoshiai et al. (Hoshiai et al., 2021) applied an intermitted 20 min (20 Hz, 4–4 s) WB-EMS protocol with “the maximum tolerable intensity” in a supine passive position for his cohort of normotensive young people. HR, BP were measured every 2 minutes during and after WB-EMS. In summary, HR, BP, but also left ventricular ejection fraction and diastolic function remained unchanged during WB-EMS and up to 10 min post-WB-EMS (Hoshiai et al., 2021).

Most researchers consider WB-EMS to be a resistance (RT) type exercise, thus one may argue that a comparison of our finding with RT studies could be legitimate. However, while WB-EMS and RT share some common characteristics, i.e., training protocols with low-volume applied with moderate to high intensity and the focus on outcomes related to muscle strength, function and mass, the principles and mechanism differ fundamentally. This is particularly the case for the high mechanical intensity of dynamic RT protocols due to exercises with external loads. Reviewing pressure response after dynamic RT (one-arm overhead press to voluntary fatigue) in novice weight-trained normotensive individuals, Fleck et al. (Fleck and Dean, 1987) reported an average increase of 87 mmHg for both systolic and diastolic BP at 70% 1RM. Even the 50% 1RM protocol triggers an average pressor response to 178/142 mmHg. However, none of the reasons listed for the very pronounced rises in BP during heavy weight lifting exercises (MacDougall et al., 1985; Palatini et al., 1989) apply to WB-EMS. This is especially true for the mechanical compression of blood vessels (Hoshiai et al., 2021) and the pronounced increase in intra-thoracic and intra-abdominal pressures (Palatini et al., 1989) through the Valsalva maneuver, “unavoidable when lifting heavy loads” (i.e., >80% 1RM) (Hackett and Chow, 2013). In contrast to RT, the non-superimposed WB-EMS protocols applied by most commercial and scientific settings involve no relevant additional load and focus on low-intensity movements/exercises with short impulse phases of 4–6 s intermitted by 4 s of rest. Another contributing aspect to BP increases is the ratio of isometric vs. isotonic components with stronger blood pressure responses applying predominantly isometric contraction (Wonisch et al., 2012). Although a short phase (<1 s) of conscious isometric tension is recommended immediately before the onset of the WB-EMS impulse, this stimulus is hardly comparable to pronounced isometric RT (Coneglian et al., 2023). In parallel, the albeit not undisputed finding that involving larger muscle groups elicits greater BP responses (Coneglian et al., 2023), i.e., an aspect particularly relevant for WB-EMS, should be considered in the context of other RT-related mechanisms and might be thus of minor relevance when applying WB-EMS as such.

Our study has several particularities and limitations that prevent ultimate evidence of an absence of negative effects of WB-EMS on MAP (and HR). First, we might have failed to determine peak BP during WB-EMS due to our non-continuous, phygmomanometric BP assessment (only) immediately pre- and post-exercise. However, the rapid HR and BP decreases reported after RT [e.g., (MacDougall et al., 1985; Lassing et al., 2023)] cannot be applied to WB-EMS. Thus we think that the “afterload blood pressure” determined in the present study might widely reflect MAP values during WB-EMS.

Missing data (n = 4) were imputed by multiple imputation. We are aware that we could omit the multiple imputation and that linear-mixed effects models offer a valid approach under the missing at random (MAR) assumption. However, we are not aware of a result clearly indicating which of the two approaches performs better in practical applications. Hence, based on our experience, we decided to use the multiple imputation approach. Further, we focus on novice WB-EMS applicants unfamiliar with complying properly with the specified stimulus intensities via RPE due to limited body awareness and reference. Indeed, the reliable regulation of stimulus intensity by RPE can be considered as a weak point of WB-EMS, particular in the early stage of application. This was confirmed by the finding that WB-EMS induced severe rhabdomyolysis was predominantly reported in novice applicants [review in (Kemmler and von Stengel, 2019)]. This made it all the more important to focus on this early phase of WB-EMS application, although a longer monitoring with evaluations of stimulus intensities in the target range of non-athletic WB-EMS application [i.e., RPE 7–8 (Kemmler et al., 2023)] might have provided additional evidence for the cardiovascular burden of WB-EMS. Another aspect not intended to be addressed by the present work was untreated hypertension. Due to the low number of untreated hypertensive participants (n = 2), we are unable to reliably address differences in blood pressure kinetics in treated vs. non-treated participants. As stated, untreated hypertension is an absolute contraindication for non-medical WB-EMS applications (Kemmler et al., 2019), but the present work provides no reliable evidence for revising this categorization. Although we do not detect different results for men vs. women, the heterogeneity of the study cohort with respect to gender and age aggravates the interpretation of our results. Finally, the formula used to calculate the MAP might have slightly confounded our result due to the underrepresentation of the more pronounced systolic BP rise particularly at increased HR. However, considering the average HR (≈100 bpm, Table 2) even after the “advanced impulse intensity” session, we conclude that this aspect does not impact our finding of low BP increases after WB-EMS. Nevertheless, we feel that it is justified to widely generalize our finding to the large cohort of middle-aged to older people, be it without or with (treated) hypertension, who may consider WB-EMS at a training option. Having said this, our finding relates to the predominately-applied intermitted WB-EMS with low-intensity movements that respect current guidelines for WB-EMS application (Kemmler et al., 2023).

In summary, although ultimate evidence might not be fully provided by the present study, we conclude that treated hypertension should not be considered as a definite barrier for WB-EMS application in moderately old to older cohorts in general. This does not include superimposed WB-EMS application that relies on high (voluntary) intensity muscle activation by moderate-high mechanical loading, superimposed by additional WB-EMS application, however.

## Data Availability

The raw data supporting the conclusion of this article will be made available by the authors, without undue reservation.

## Footnotes

1 Combined with easy movements

2 Apart from the willingness for WB-EMS training, no further eligibility criteria were applied

3 I.e., Hypertension: systolic BP (SBP) ≥ 140 mmHg or diastolic BP (DBP) ≥ 90 mmHg or antihypertensive treatment

4 Unlike superimposed WB-EMS application, this approach focuses on NEMS effects with a minor contribution from voluntary muscle activation

5 Due to differences in sensitivity, it is not possible to determine whether the absolute intensity corresponds properly to the specified RPE

6 This refers to the main controller of the device. 100% constitutes the maximum device performance

7 Values refer to normotensive participants; maximum MAP in the hypertensive subgroup was 124 mmHg, i.e. 100 mmHg diastolic BP and 174 mmHg systolic BP

8 MAP results for the total group were presented in Figure 2.
